# Examining Stroke Disparities in Florida: Relationships Among County Classification, Age-Adjusted Stroke Mortality Rates, and the Presence of Primary Stroke Centers

**DOI:** 10.5888/pcd18.200561

**Published:** 2021-06-10

**Authors:** Daudet Ilunga Tshiswaka, Colleen Murphy, Guy-Lucien Whembolua, Olajide Williams

**Affiliations:** 1 University of West Florida, Department of Public Health, Pensacola, Florida; 2 University of Cincinnati, Department of Africana Studies, Cincinnati, Ohio; 3 Columbia University, Department of Neurology, New York, New York

**Figure:**
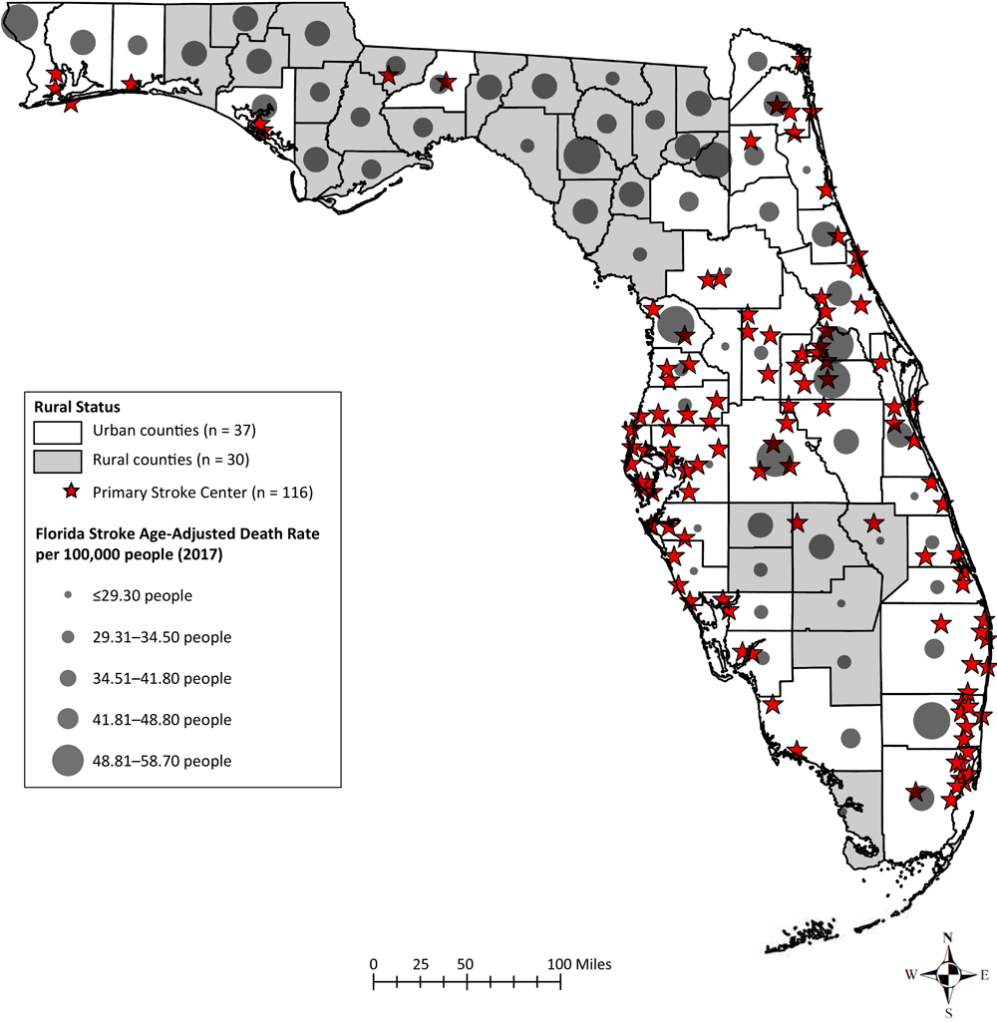
Static display of Florida’s distribution of rural versus urban and high versus low death rate, where stroke centers are in relation to urban versus rural and low versus high primary stroke centers, and age-adjusted stroke mortality rates, by quintile, in urban versus rural counties in 2017. Data sources: Florida’s Geospatial Open Data Portal 2017, 2018; Rural Health Information Hub 2017; US Census Bureau, 2010; Florida Geographic Data Library, 2012.

## Background

Although overall stroke mortality has declined in the United States for decades, recent data show that this decline in stroke deaths has slowed and that stroke remains 1 of the leading causes of death at the state level (1). In Florida, stroke is the fifth leading cause of death and was responsible for 12,602 deaths in 2017. Florida’s death rate is 38.9 per 100,000 population and, in 2021, it is tied with Illinois at 20th place in stroke-related death rate rankings by state (2).

As part of an effort to improve the quality of care provided to stroke patients, primary stroke centers were created with a strict set of criteria for certifying hospitals that meet predefined standards (3) with the goal of stabilizing and providing emergency care for acute stroke patients (4). With these goals in mind, a patient is admitted to a primary stroke center or a comprehensive stroke center based on the severity of stroke symptoms. Although comprehensive stroke centers are equipped to provide care for complex stroke patients who often have more advanced therapeutic needs, primary stroke centers are equipped to provide care for less complex stroke patients and can administer acute stroke thrombolysis in a timely manner.

Having limited or no access to stroke centers remains a major challenge for many stroke patients. In the US, the scarcity of stroke centers is more pronounced in rural areas (5). In Florida, a rural county is a county with either 1) a population of 75,000 people or less, or 2) a population of less than 125,000 people and contiguous with a county that has a population of less than 75,000 people (6). By this definition, 30 out of the 67 counties in Florida are rural (7) and they contain 8.8% of Florida’s population (8). Considering the importance of stroke centers, a gap exists in the literature assessing the relationship between county classification, age-adjusted stroke mortality rates, and the number of primary stroke centers in Florida.

The purpose of our research was to create maps that illustrate the relationship between age-adjusted stroke mortality rates and the presence of primary stroke centers in Florida. We hypothesized that stroke mortality will be higher in regions of Florida with fewer primary stroke centers.

## Data and Methods

We used publicly available age-adjusted stroke mortality data for 2017 from the Florida Department of Health Death Data Viewer (9). The 2017 primary stroke center shapefiles and the Florida county lines came from the Florida Geographic Data Library and the ArcGIS Hub (7,10,11). The US Census Bureau website provided information about urban and rural counties as of 2010 (12). Independent variables were rural (n = 30) and urban (n = 37) county status, and dependent variables were number of primary stroke centers (n = 116) and age-adjusted stroke mortality rates in Florida.

We geocoded primary stroke centers by using the Florida county lines shapefile as the basis for locating and indicating exact primary stroke centers onto the map (13). The Capital Regional Medical Center — Gadsden Memorial campus (in Gadsden County) was matched with zip code 32351 rather than 32353, as shown in the list of primary stroke centers. Bartow Regional Medical Center in Polk County was also changed from zip code 33831 to 33830. We used ArcMap 10.3 (ESRI) for geocoding and mapping purposes (13).

Point-biserial correlations were performed to determine the correlation between the urban county versus rural county status and age-adjusted stroke mortality rates. The test for normality (ie, Shapiro-Wilk test) suggested that age-adjusted stroke mortality rates were normally distributed throughout Florida’s urban and rural counties (*P* > .05). The number of primary stroke centers across Florida, however, was not normally distributed (*P* < .05); therefore, we performed a nonparametric test (ie, Mann-Whitney *U* test) to consider the nonnormal distribution of urban county versus rural county primary stroke centers throughout Florida. More precisely, the Mann-Whitney *U* test was used to determine whether the number of primary stroke centers differed in urban and rural counties. All statistical analyses were performed using SPSS version 24 (IBM Corp).

## Highlights

Geocoding indicated that 116 primary stroke centers were primarily in the west, central, and east regions of the state. The point-biserial correlation coefficient for the relationship between urban counties versus rural counties and age-adjusted stroke mortality rates was *r* = 0.05, although the relationship was not significant (*P* = .67). In the Mann-Whitney *U* test, the number of primary stroke centers in urban counties (mean = 47.8 centers, n = 37 counties) was significantly higher than the number in rural counties (mean = 17.0 centers, n = 30 counties) (Mann-Whitney *U* = 45, *P* < .001.)

## Action

Analyzing the relationship between county classification, age-adjusted stroke mortality rates, and primary stroke centers has implications for stroke system development at the state level. Our distribution map indicates a primary stroke center disparity in Florida, favoring urban counties with more primary stroke centers than rural counties. This finding underscores the need for more equitable resource allocation regarding primary stroke center availability in Florida.

The use of telemedicine for the treatment of stroke (ie, telestroke) may help reduce primary stroke center disparity by helping rural hospitals meet eligibility for certification as a hospital for treating acute stroke (14). Telestroke is a promising strategy for addressing the acute management of stroke patients, and barriers related to telestroke reimbursement have been addressed by passage of the Furthering Access to Stroke Telemedicine Act. Constraints to the use of telestroke, however, include the availability and affordability of technology, the need for ongoing technological support, logistical challenges related to the potential need for examination assistance by a participating bedside clinician or nurse, and several legal and ethical questions about provider credentials and patient safety and privacy (15,16).
